# Staphylococcal LTA antagonizes the B cell-mitogenic potential of LPS

**DOI:** 10.1038/s41598-018-19653-y

**Published:** 2018-01-24

**Authors:** Seok-Seong Kang, Sun Kyung Kim, Jung Eun Baik, Eun Byeol Ko, Ki Bum Ahn, Cheol-Heui Yun, Seung Hyun Han

**Affiliations:** 1 Department of Food Science and Biotechnology, Dongguk University-Seoul, Goyang, 10326 Republic of Korea; 2 Department of Oral Microbiology and Immunology, DRI, and BK21 Plus Program, School of Dentistry, Seoul National University, Seoul, 08826 Republic of Korea; 3 Department of Agricultural Biotechnology and Research Institute for Agriculture and Life Sciences, Seoul National University, Seoul, 08826 Republic of Korea

## Abstract

Lipoteichoic acid (LTA) of Gram-positive bacteria is regarded as the counterpart biomolecule of lipopolysaccharide (LPS) of Gram-negative bacteria because of their structural and immunological similarities. Although LPS induces a strong polyclonal expansion of B cells, little is known about the effect of LTA on B cell proliferation. In the present study, we prepared LTAs from Gram-positive bacteria and examined their effect on splenic B cell proliferation. Unlike LPS, LTA did not induce B cell proliferation. Instead, *Staphylococcus aureus* LTA (Sa.LTA) appeared to inhibit LPS-induced B cell proliferation *in vitro*, *ex vivo*, and *in vivo* models. Such effect was observed neither in splenocytes from Toll-like receptor 2 (TLR2)-deficient mice nor in the purified splenic B cells. Furthermore, decreased ERK phosphorylation appeared to be responsible for this phenomenon. Collectively, our results support that Sa.LTA inhibited LPS-induced B cell proliferation through the decrease of ERK phosphorylation via TLR2 signaling pathway.

## Introduction

Microbial products often lead to polyclonal expansion of B cells and differentiation of antibody-secreting cells, which play a central role in humoral adaptive immunity^1^. The expansion of B cells can be induced by thymus-dependent (Td) or -independent (Ti) antigens^2^. Td antigens are mostly soluble proteins or peptides recognized by B cell receptors (BCR). They are processed by antigen-presenting cells and presented in association with MHC class II molecules to T helper cells^3^. Td antigens are unable to directly induce polyclonal expansion of B cells in the absence of cognate interaction with effector T helper cells^4^. Ti antigens are further classified into type I and type II antigens. Type I Ti antigens, such as bacterial lipopolysaccharide (LPS), possess B cell mitogenic activity, which induces polyclonal expansion of B cells^5^. Type II Ti antigens such as polysaccharides of *Streptococcus pneumoniae* with repeating units directly activate B cells by cross-linking BCRs in a multivalent fashion^4^. However, unlike type I Ti antigens, type II Ti antigens have no B cell mitogenic activity.

LPS induces expansion of B cells through the interaction with Toll-like receptor 4 (TLR4)/MD-2 complex. LPS can directly bind to MD-2 and promote biological activity through TLR4^6^. RP105 is considered an additional LPS receptor on B cells that is strictly associated with MD-1^7^. It is known that B cells lacking RP105 or MD-1 have impaired LPS-induced B cell proliferation^7^. In addition, LPS promotes B cell proliferation through the activation of accessory cells such as macrophages by inducing secretion of B cell-activating factors^8^. Negative regulatory mechanisms involved in the inhibition of B cell proliferation have been suggested. For example, inhibition of B cell proliferation is caused by up-regulation of perforin and granzyme in regulatory T cells when B cells are co-cultured with CD4^+^CD25^+^ T cells and LPS^9^. IL-10 and TGF-β also inhibit LPS-induced B cell proliferation^10,11^. Although the role of IL-27 in cell proliferation remains ambiguous, IL-27 is involved in suppressing proliferation of cells such as T cells and lymphatic endothelial cells^12,13^.

Gram-positive bacteria express lipoteichoic acid (LTA) which is analogous to LPS with respect to structural and immunological characteristics^14,15^. Both LPS and LTA are amphiphilic complex molecules consisting of hydrophobic glycolipids and hydrophilic polysaccharides^14^. They induce various pro-inflammatory cytokines and chemokines^15^. Although both LTA and LPS share similar structural and immunological characteristics, they have distinctive properties on their immunological and pathophysiological roles. For example, LTA is recognized by TLR2 and triggers a cell signaling cascade through MyD88-dependent pathway^16^, whereas LPS recognized by TLR4 triggers downstream signaling via MyD88-dependent and TRIF-dependent pathways^16,17^. LPS is a powerful agent that can provoke inflammatory responses, whereas LTA exhibits relatively weak induction of inflammatory responses that can be amplified in the presence of other bacterial components such as peptidoglycan^18^.

Although LTA has been considered the counterpart of LPS, the mitogenic potential of LTA on B cells has not yet been fully defined; however, LPS has been extensively investigated as a potent B cell mitogen. Furthermore, LTAs from various Gram-positive bacteria may induce distinct immune responses due to differences in their molecular structure^19^. Here, we prepared highly purified and structurally intact LTAs from various Gram-positive bacteria and investigated their mitogenic potential on mouse splenic B cell expansion.

## Results

### Staphylococcal LTA inhibits LPS-induced B cell proliferation

**Figure 1:**
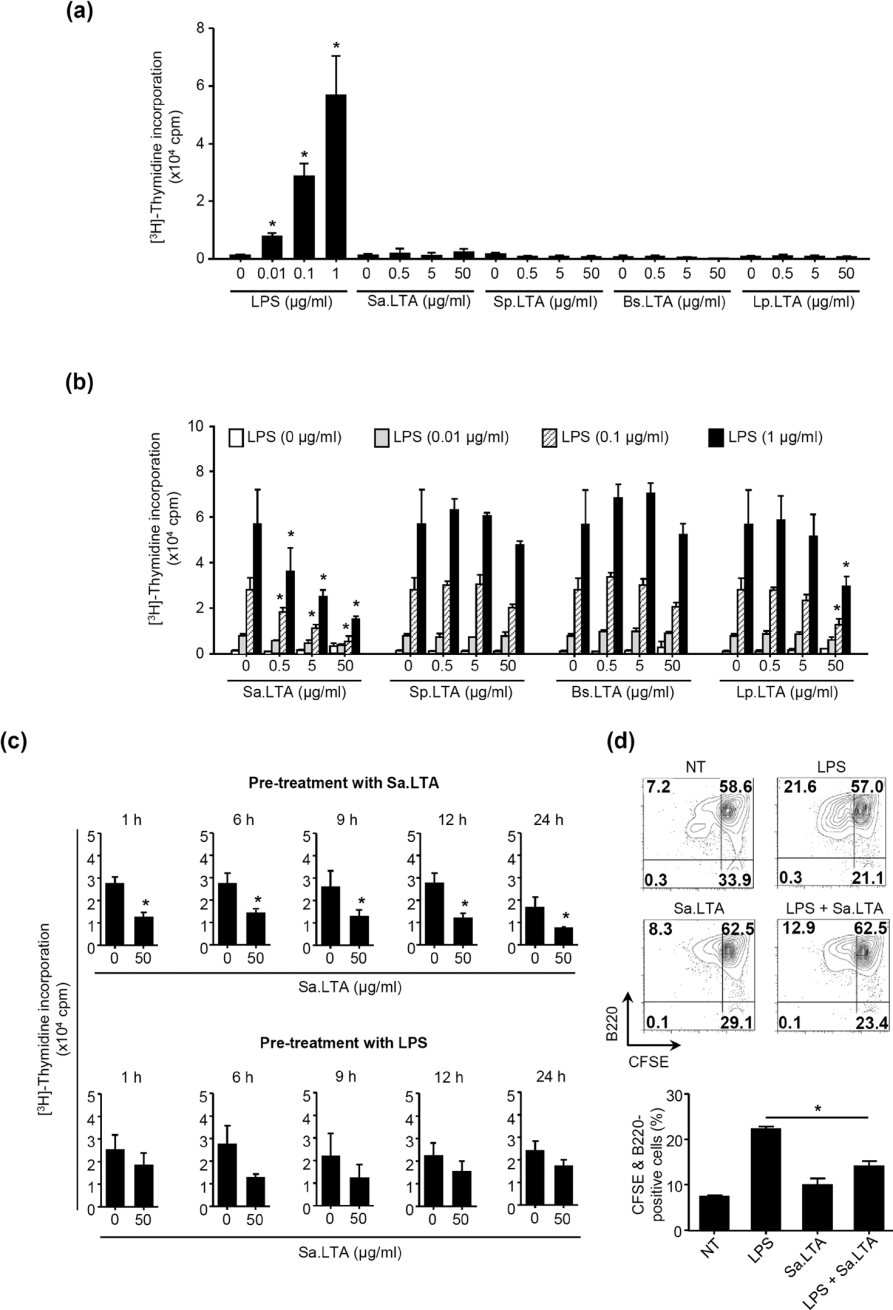
Staphylococcal LTA inhibits LPS-induced B cell proliferation. (**a**) Splenocytes (1 × 10^6^ cells/ml) were stimulated with *S*. *aureus* LTA (Sa.LTA), *S*. *pneumoniae* LTA (Sp.LTA), *B*. *subtilis* LTA (Bs.LTA), *L*. *plantarum* LTA (Lp.LTA), or *E*. *coli* LPS at the indicated concentrations for 72 h. Then, the cells were pulsed with 0.5 μCi/well of [^3^H]-thymidine for the last 6 h of culture. Cell proliferation was determined by [^3^H]-thymidine incorporation. *P < 0.05 compared with control culture without LPS stimulation. (**b**) Splenocytes were stimulated with LTAs and/or LPS at the indicated concentrations for 72 h and cell proliferation was determined as described above. *P < 0.05 compared with control culture without LTA stimulation. (**c**) Splenocytes were pre-treated with Sa.LTA (50 μg/ml) for 1, 6, 9, 12, or 24 h and subsequently stimulated with LPS for 72 h (*Upper panel*). Conversely, splenocytes were pre-treated with LPS (0.1 μg/ml) for 1, 6, 9, 12, or 24 h and then stimulated with Sa.LTA (50 μg/ml). At 72 h incubation after LPS treatment, cell proliferation was determined as described above (*lower panel*). *P < 0.05 compared with control culture without Sa.LTA stimulation. All data are expressed as the average count per minute (cpm) ± standard deviation of three separate experiments. (**d**) CFSE-labeled splenocytes (1 × 10^6^ cells/ml) were stimulated with Sa.LTA (50 μg/ml) and/or LPS (0.1 μg/ml) for 72 h. Then, the cells were stained with PerCP-conjugated rat anti-mouse CD45R/B220 antibody and B cell proliferation was determined by flow cytometry. NT denotes non-treatment.

To determine whether LTA can induce cell proliferation, we examined the proliferative ability of LTA in splenocytes. Splenocytes were stimulated with LTAs from various Gram-positive bacteria including *Staphylococcus aureus* (Sa.LTA), *S. pneumoniae* (Sp.LTA), *Bacillus subtilis* (Bs.LTA), or *Lactobacillus plantarum* (Lp.LTA) at various concentrations. Figure 1a demonstrates that none of the LTAs tested in this study induced splenocyte proliferation, whereas ultra-pure LPS from *E. coli* K12 dose-dependently and significantly induced splenocyte proliferation, implying that LTA does not affect splenocyte proliferation at all or perhaps potentially suppresses it. Thus, we further examined the effect of LTA on the LPS-induced splenocyte proliferation. Interestingly, Sa.LTA substantially inhibited LPS-induced splenocyte proliferation in a dose-dependent manner (Fig. 1b). In contrast to the inhibitory effect of Sa.LTA, except for a slight inhibitory effect by Lp.LTA at high concentration, the other LTAs hardly inhibited LPS-induced splenocyte proliferation (Fig. 1b). Thus, Sa.LTA was used for the rest of experiments. Next, to examine whether pre- or post-treatment with LTA would have different effects on the proliferative response, splenocytes were pre-treated with Sa.LTA for 1, 6, 9, 12 or 24 h and subsequently treated with LPS or vice versa. The proliferative response was then determined at 72 h after LPS treatment. Similar to co-treatment with Sa.LTA and LPS, pre-treatment with Sa.LTA exhibited potent inhibition of the LPS-induced proliferative response regardless of the duration of Sa.LTA pre-treatment (Fig. 1c, *upper panel*). However, post-treatment with Sa.LTA resulted in only partial inhibitory activity (Fig. 1c, *lower panel*), suggesting that pre-treatment with LTA is superior to post-treatment for its inhibitory effect. Since LPS is a well-known murine B cell mitogen^5^, we examined whether the decreased proliferative response was due to selective inhibition of B cell proliferation. As shown in Fig. 1d, LPS-induced B cell proliferation was diminished in the presence of Sa.LTA, demonstrating that Sa.LTA inhibited LPS-induced B cell proliferation. Although Sa.LTA inhibited bacterial lipoprotein-induced B cell proliferation, the inhibitory level was relatively moderate (data not shown).

### Sa.LTA-mediated inhibitory effect of B cell proliferation is involved in TLR2

**Figure 2:**
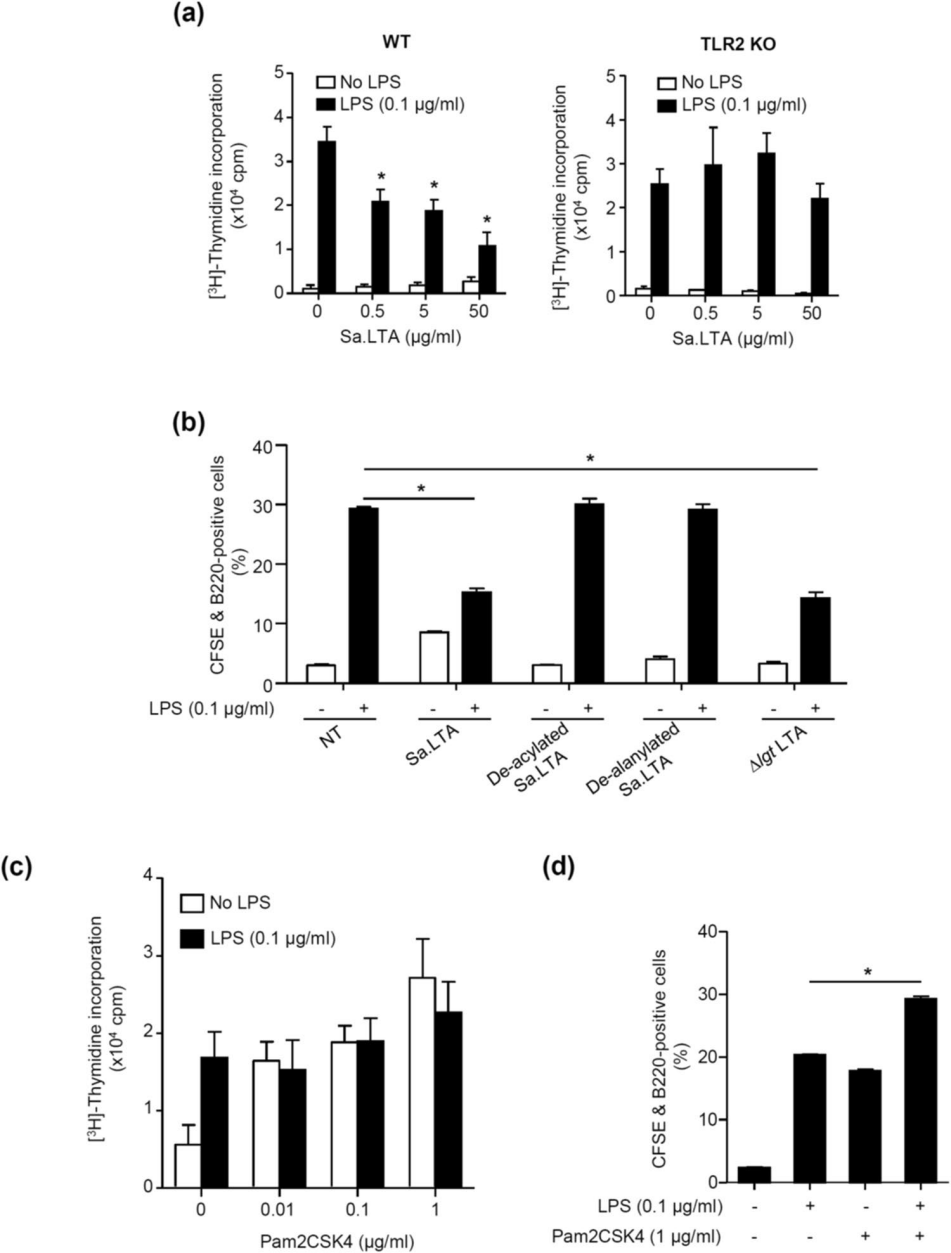
Sa.LTA-mediated inhibitory effect of B cell proliferation is involved in TLR2. (**a**) Splenocytes (1 × 10^6^ cells/ml) were isolated from wild-type or TLR2-deficient C57BL/6 mice and stimulated with the indicated concentrations of Sa.LTA and/or LPS for 72 h. Then, cell proliferation was determined by [^3^H]-thymidine incorporation. Data are expressed as the average cpm ± standard deviation of three separate experiments. *P < 0.05 compared with LPS treatment group without Sa.LTA. (**b**) CFSE-labeled splenocytes (1 × 10^6^ cells/ml) were incubated with de-acylated LTA, de-alanylated LTA or LTA from the *S. aureus* Δ*lgt* strain (50 μg/ml each) in the absence or presence of LPS (0.1 μg/ml) for 72 h. Then, the cells were stained with PerCP-conjugated rat anti-mouse CD45R/B220 antibody and B cell proliferation was determined by flow cytometry. NT denotes non-treatment. (**c**) Splenocytes (1 × 10^6^ cells/ml) were stimulated with Pam2CSK4 and/or LPS at the indicated concentrations for 72 h and cell proliferation was determined by [^3^H]-thymidine incorporation. Data are expressed as the average cpm ± standard deviation of three separate experiments. *P < 0.05 compared with control culture without LPS stimulation. (**d**) CFSE-labeled splenocytes were stimulated with Pam2CSK4 (1 μg/ml) and/or LPS (0.1 μg/ml) for 72 h. Then, the cells were stained with PerCP-conjugated rat anti-mouse CD45R/B220 antibody and B cell proliferation was determined by flow cytometry.

Next, we examined whether the inhibitory effect of Sa.LTA on LPS-induced proliferation was mediated through TLR2. Sa.LTA dose-dependently inhibited LPS-induced splenocyte proliferation in wild-type mice, whereas it was not observed in splenocytes from TLR2-deficient mice. This result suggests that TLR2 is essential for the inhibitory effect of Sa.LTA on LPS-induced splenocyte proliferation (Fig. 2a). Furthermore, we examined whether the lipid moiety of Sa.LTA plays a role in the inhibitory effect, given the fact  that LTA binds to TLR2 through its lipid moiety^20^ and de-acylated LTA loses its immunostimulating potential^21^. As shown in Fig. 2b, de-acylated Sa.LTA failed to inhibit LPS-induced B cell proliferation, suggesting that the lipid moieties of Sa.LTA played a critical role in the inhibition of LPS-induced B cell proliferation. Because D-alanine in the glycerol phosphate repeating unit of the LTA structure is also critical for its immunostimulatory activity^22^, we examined whether de-alanylated Sa.LTA affected the inhibition of LPS-induced B cell proliferation. De-alanylated Sa.LTA did not inhibit LPS-induced B cell proliferation (Fig. 2b), indicating that D-alanine of Sa.LTA structure is also crucial for the inhibition of LPS-induced B cell proliferation. Hashimoto *et al*. suggested that the lipoproteins present as an impurity in Sa.LTA preparation, but not LTA itself, would be principal immunostimulatory molecules^23^. Therefore, we examined whether LTA from lipoprotein-deficient *S. aureus* (Δ*lgt*) confers inhibitory potential to LPS-induced B cell proliferation. LTA from the Δ*lgt* strain also inhibited LPS-induced B cell proliferation close to that of LTA from wild-type *S. aureus* (Fig. 2b). Furthermore, to investigate whether TLR2 ligands commonly inhibit LPS-induced proliferation, we used another TLR2 ligand, Pam2CSK4, a synthetic lipopeptide known to mimic Gram-positive bacterial lipoproteins^24^. Pam2CSK4 sufficiently induced splenocyte proliferation in a dose-dependent manner and, unlike Sa.LTA, Pam2CSK4 failed to inhibit LPS-induced splenocyte proliferation (Fig. 2c). Although a low concentration of Pam2CSK4 (*e.g*. 0.01 μg/ml) induced much higher proliferative response than LPS (Fig. 2c), the proliferative response was considerably increased by LPS at a higher concentration (1 μg/ml) (Fig. 1a). Notably, Pam2CSK4 alone directly elicited B cell proliferation and even further enhanced LPS-induced B cell proliferation (Fig. 2d). These results suggest that Sa.LTA preferentially inhibited LPS-induced proliferative responses.

### Sa.LTA strongly inhibits LPS-induced B cell proliferation in an indirect manner through interaction with accessory cells

**Figure 3:**
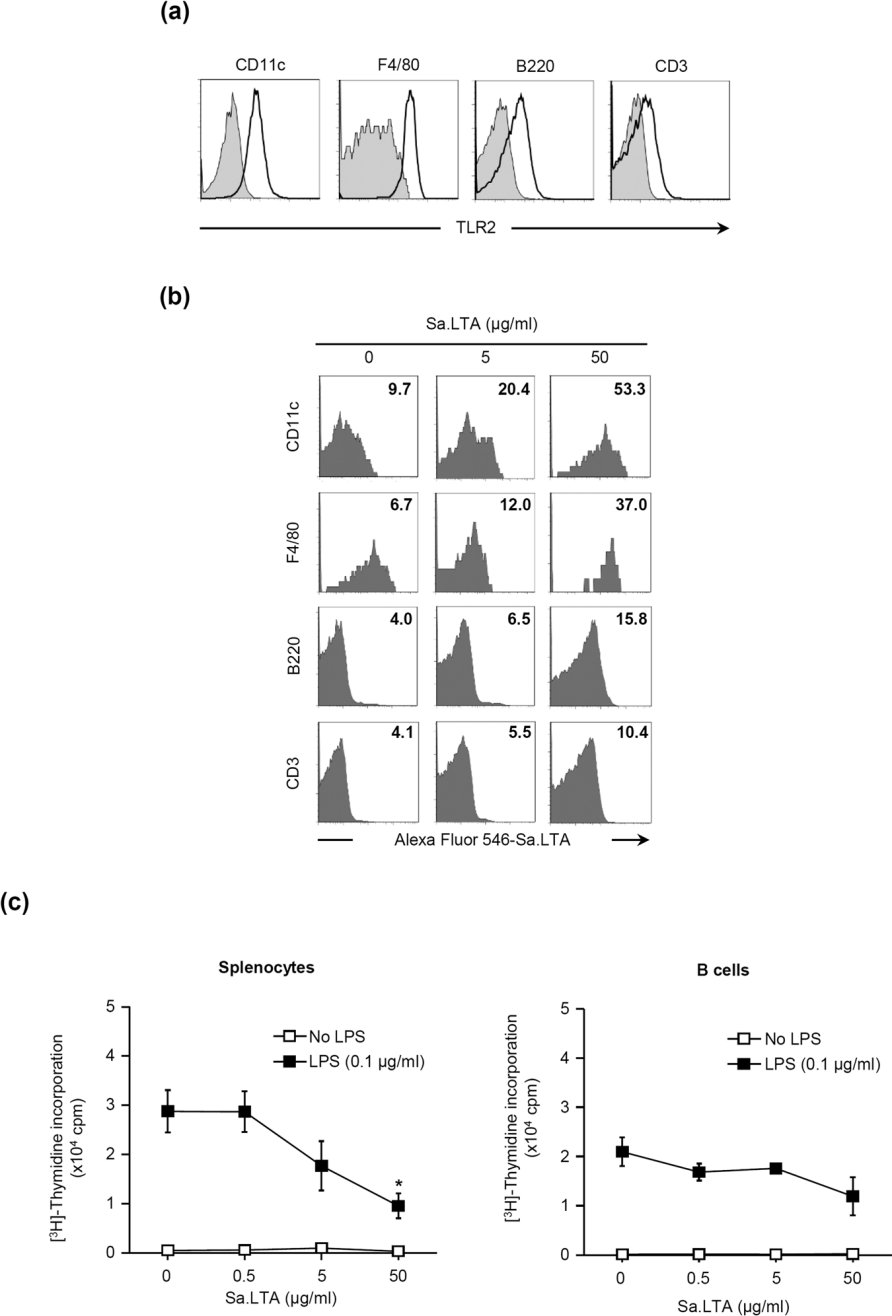
Staphylococcal LTA inhibits LPS-induced B cell proliferation through interaction with accessory cells in an indirect manner. (**a**) Splenocytes were stained with PE-conjugated rat anti-mouse TLR2 antibody and TLR2 surface expression on gated CD11c^+^, F4/80^+^, B220^+^, or CD3^+^ cells was analyzed by flow cytometry. The gray-filled area indicates histograms of the isotype control. One representative result from three similar results is shown. (**b**) Splenocytes were incubated with Alexa Fluor^®^ 546-conjugated Sa.LTA for 1 h at 4 °C and the binding of Sa.LTA to CD11c^+^, F4/80^+^, B220^+^, or CD3^+^ cells was analyzed by flow cytometry. The number indicates mean fluorescence intensity (MFI). One representative result from three similar results is shown. (**c**) Splenic B cells were negatively purified through magnetic cell sorting as described in the Materials and Methods. Whole splenocytes or purified B cells were incubated with Sa.LTA and/or LPS at the indicated concentrations for 72 h and cell proliferation was determined by [^3^H]-thymidine incorporation. Data are expressed as the average cpm ± standard deviation of three separate experiments. *Indicates *P* < 0.05 compared with LPS treatment group without Sa.LTA.

Since polyclonal B cell proliferation is regulated by accessory cells such as macrophages^25^, we investigated the interaction of Sa.LTA with various cell types. First, we determined TLR2 expression on the surface of various cell types of splenocytes. TLR2 was expressed on CD11c^+^, F4/80^+^, B220^+^, or CD3^+^ cells. In particular, CD11c^+^ and F4/80^+^ cells, which are mainly dendritic cells and macrophages, showed relatively higher expression (Fig. 3a). Next, Sa.LTA conjugated with Alexa Fluor^®^ 546 was used to examine whether Sa.LTA interacted with each cell type. Total splenocytes treated with Sa.LTA conjugated with Alexa Fluor^®^ 546 including lymphocytes, monocytes and granulocytes were gated based on side and forward scatters and then, CD11c^+^, F4/80^+^, B220^+^, or CD3^+^ cells were analyzed. The results showed a higher binding of Sa.LTA to CD11c^+^ and F4/80^+^ cells over CD3^+^ and B220^+^ cells (Fig. 3b). Accordingly, we examined whether Sa.LTA indirectly inhibited LPS-induced B cell proliferation. The results showed that the inhibitory effect of Sa.LTA on the proliferative responses was less effective in purified B cells than in total splenocytes (Fig. 3c). Sa.LTA weakly inhibited LPS-induced B cell proliferation without accessory cells. However, Sa.LTA had a greater inhibitory effect in the presence of accessory cells, implying that Sa.LTA may indirectly inhibit LPS-induced B cell proliferation with the help of accessory cells.

### Sa.LTA inhibits LPS-induced B cell proliferation by decreasing ERK phosphorylation

**Figure 4:**
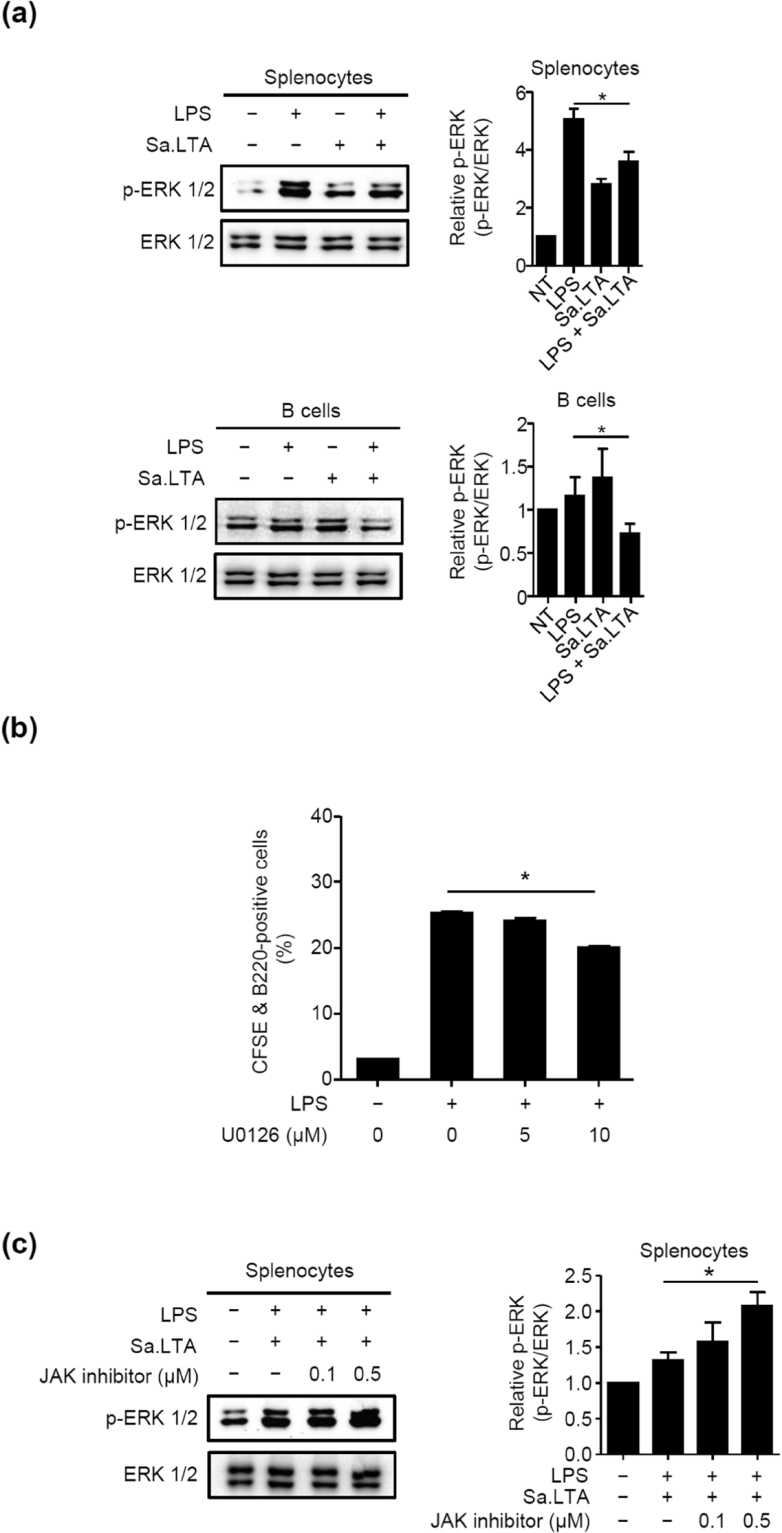
Staphylococcal LTA inhibits LPS-induced B cell proliferation by decreasing ERK phosphorylation. (**a**) Splenocytes or purified B cells (2.5 × 10^7^ cells/ml) were stimulated with LPS (0.1 μg/ml) and/or Sa.LTA (50 μg/ml) for 24 h. After stimulation, the cells were lysed and equal amounts of proteins were subjected to Western blot analysis using antibodies specific to the non-phosphorylated or phosphorylated form of ERK. (**b**) CFSE-labeled splenocytes (1 × 10^6^ cells/ml) were pre-treated with an ERK-specific inhibitor, U0126 (5 or 10 μM), for 1 h and subsequently stimulated with LPS (0.1 μg/ml) for 72 h. After stimulation, the cells were stained with PerCP-conjugated rat anti-mouse CD45R/B220 antibody and B cell proliferation was determined by flow cytometry. (**c**) Splenocytes (2.5 × 10^7^ cells/ml) were pre-treated with a JAK inhibitor (0.1 or 0.5 μM) for 1 h and subsequently stimulated with Sa.LTA (50 μg/ml) and/or LPS (0.1 μg/ml) for 24 h. After stimulation, the cells were lysed and equal amounts of proteins were subjected to Western blot analysis using antibodies specific to the non-phosphorylated or phosphorylated forms of ERK. The gels were run under the same experimental conditions and the blots were representative of three independent experiments. The results were shown as cropped blots (Whole blots are shown in Supplementary Figure S1). Relative p-ERK values were obtained by the densitometric analysis of Western blot data from three independent experiments.

It has been established that sustained ERK activation is associated with B cell proliferation^26^. Thus, we determined ERK phosphorylation using Western blot analysis. A significant decrease of LPS-induced ERK phosphorylation was observed in the presence of Sa.LTA in both splenocytes and B cells (Fig. 4a). Additionally, splenocytes treated with an ERK-specific inhibitor, U0126, resulted in the inhibition of LPS-induced proliferation (Fig. 4b). Inhibition of cell proliferation is associated with sustained JAK/STAT1 activation, which regulates the transcription of genes involved in cell cycle control^27^. A JAK inhibitor diminished the inhibitory potential of Sa.LTA on LPS-induced B cell proliferation (data not shown). Besides, enhanced ERK phosphorylation was also observed in splenocytes treated with LPS and Sa.LTA after the pretreatment with the JAK inhibitor (Fig. 4c). Collectively, Sa.LTA led to the inhibition of LPS-induced B cell proliferation by decreasing ERK phosphorylation.

### Sa.LTA negatively affects LPS-induced B cell proliferation ***in vivo*** and ***ex vivo***

**Figure 5:**
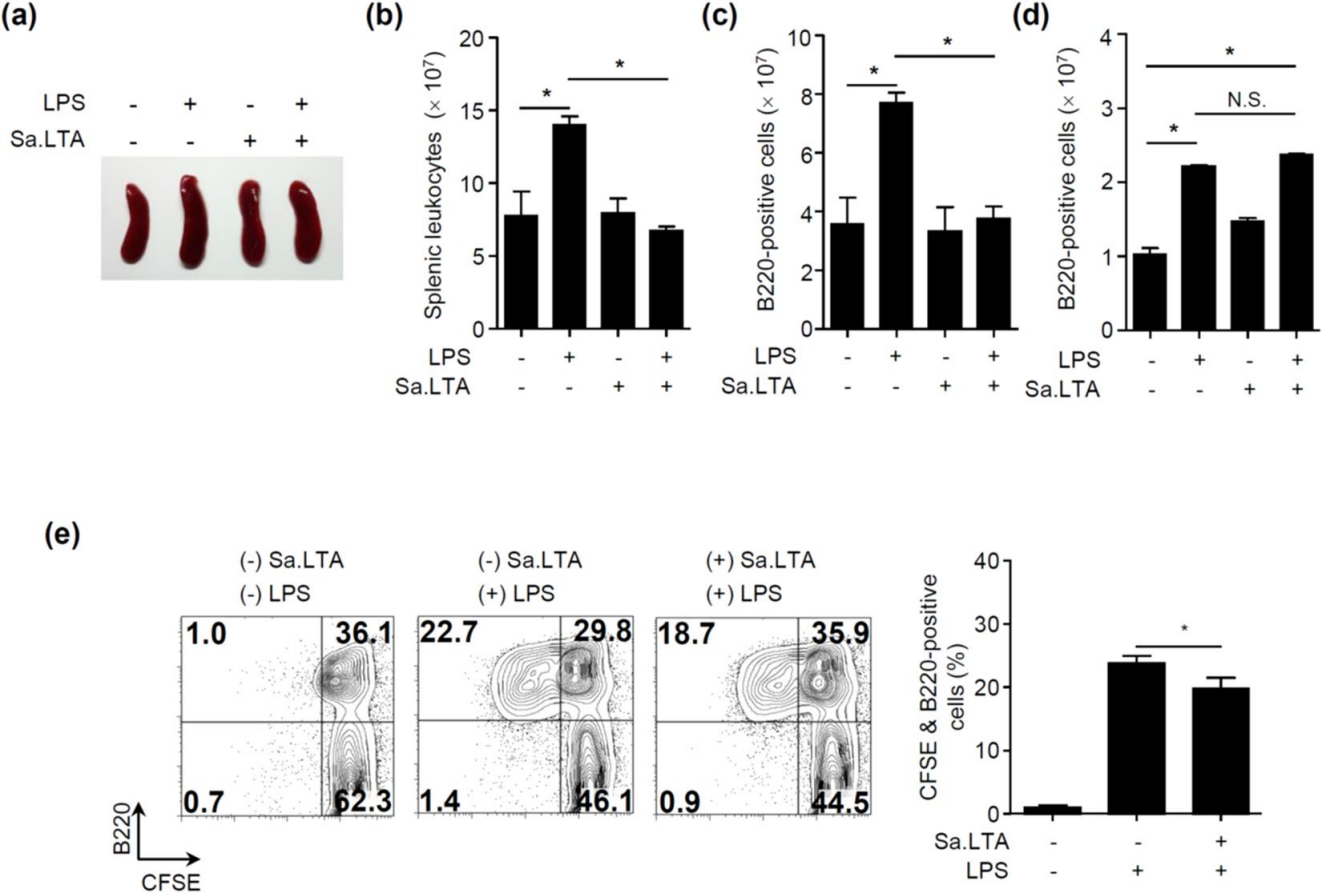
Staphylococcal LTA inhibits LPS-induced B cell proliferation *in vivo*. C57BL/6 mice (n = 3 per group) were *i.p*. injected with LPS (50 μg/mouse) and/or Sa.LTA (300 μg/mouse). After 48 h, spleens were photographed (**a**) and splenic leukocytes (**b**) and B220^+^ cells (**c**) were quantified using flow cytometric analysis. (**d**) TLR2-deficient C57BL/6 mice (n = 3 per group) were *i.p*. injected with LPS (50 μg/mouse) and/or Sa.LTA (300 μg/ml). After 48 h, spleens were removed and distribution of B220^+^ cells were analyzed using flow cytometric analysis. Data are expressed as the mean value ± standard deviation of results from three mice. (**e**) Mice (n = 3 per group) were *i.p*. injected with Sa.LTA (100 μg/mouse). After 24 h, spleens were removed and single cell suspensions were prepared as described in the Materials and Methods. Splenocytes were CFSE-labeled and stimulated with LPS (0.1 μg/ml) for 72 h. After stimulation, cells were stained with PerCP-conjugated rat anti-mouse CD45R/B220 antibody and B cell proliferation was determined by flow cytometry. The number indicates the percentage of cells in the designated areas. N.S. denotes not significant.

We confirmed the inhibitory effect of Sa.LTA on LPS-induced B cell proliferation *in vivo*. Mice given LPS alone by *i.p*. injection developed splenomegaly; however, the degree of splenomegaly was reduced when mice were co-administered with Sa.LTA and LPS at 48 h (Fig. 5a). Furthermore, the increased number of leukocytes and B cells in spleens were reduced when mice were co-administered with Sa.LTA and LPS (Fig. 5b and c, respectively). In addition, Sa.LTA mediated the inhibitory effect on LPS-induced B cell proliferation through TLR2. Figure 5d illustrated that LPS treatment elicited B cell proliferation in splenocytes whereas the inhibitory effect of LPS-induced B cell proliferation was not observed in splenocytes from TLR2-deficient mice given Sa.LTA. Moreover, to access whether LTA prevents excessive lymphocyte proliferation during the infection, mice were injected *i.p*. with Sa.LTA and subsequently spleens were removed and stimulated with LPS. As shown in Fig. 5e, the proliferative response was diminished in B cells of splenocytes derived from mice given Sa.LTA compared with B cells of splenocytes derived from mice not given Sa.LTA. Notably, Sa.LTA diminished LPS-induced splenic B cell proliferation by 18% *ex vivo* while Sa.LTA inhibited LPS-induced splenic B cell proliferation by 30% *in vitro*. Although LTAs from non-pathogenic or beneficial bacteria need to be applied, it can be assumed that pretreated LTA may prevent excessive lymphocyte proliferation. These results confirm an inhibitory effect of Sa.LTA on LPS-induced B cell proliferation *in vivo* and *ex vivo*.

## Discussion

Bacterial antigens triggering T cell-independent immune responses can be classified into two types; type I antigens including LPS, peptidoglycans and lipoproteins have polyclonal B cell mitogenic activity^28,29^ while type II antigens including most bacterial polysaccharides have no polyclonal B cell mitogenic activity, but do exhibit monoclonal B cell proliferative potential^30^. Our findings demonstrate that LTA alone has no mitogenic ability on B cells, and, thus, it can be excluded in the classification of type I Ti antigens. Surprisingly, Sa.LTA elicits an antagonistic effect on LPS-induced B cell proliferation.

Although these LTAs increased TLR2 activation and LTAs from *S. aureus* and *B. subtilis* substantially induced the production of nitric oxide and pro-inflammatory cytokines such as IL-6 and TNF-α^19,31^, LTA alone did not induce B cell proliferation. Among the bacterial LTAs tested in this study, Sa.LTA only showed a significant antagonistic effect on LPS-induced B-cell proliferation. This result can be possibly explained by the heterogeneity found in the LTA structure of various Gram-positive bacteria. LTA is an amphiphile consisting of hydrophilic polysaccharides and hydrophobic lipid moieties^14^. LTAs of most Gram-positive bacteria including Sa.LTA, Bs.LTA, and Lp.LTA have polyglycerol phosphate-type polysaccharides, whereas Sp.LTA has polyribitol phosphate-type polysaccharides^32,33^. Moreover, the D-alanine content of polyglycerol phosphates varies, which is known to affect the immunological properties of LTAs^34^. In fact, Sa.LTA contains approximately 70% D-alanine, whereas Bs.LTA and Lp.LTA have 25% and 41.7% D-alanine, respectively^34,35^. Our observation indicated that high D-alanine content of Sa.LTA showed the most effective inhibition of B cell proliferation whereas the deficiency of D-alanine failed to inhibit B cell proliferation. A previous study has suggested that TLR2 has a hydrophobic pocket interacting with lipid chains, indicating that the lipid moieties of Sa.LTA are associated with TLR2 engagement^20^. Although it is unclear that D-alanine of LTA structure interacts with TLR2-ligand binding site, several evidence showed that the loss of D-alanine failed to induce immune responses through TLR2^19,34^. Therefore, both lipid moieties and D-alanine content are essential for the inhibitory effect of B cell proliferation. LTAs used in early studies have been often contaminated with unwanted endotoxins and structural damages due to the use of a strong organic solvent such as phenol^36,37^, leading to unexpected or erroneous results were obtained by LTAs. However, advanced methods such as the extraction with a mild organic solvent such as butanol and serial chromatographies with hydrophobic interaction and ion-exchange column have been recently employed to purify intact LTAs with structural and immunological properties^19^. With respect to their lipid moieties, Sa.LTA has diacyl chains (two saturated), Lp.LTA has triacyl chains (two saturated and one unsaturated), and Sp.LTA has diacyl chains (one saturated and one unsaturated)^38,39^. Remarkably, lipidated molecules with a higher number of saturated fatty acids might favorably interact with lipid rafts clustering in the TLR2-signaling complex on the surface of the cell membrane^20^.

It has been shown that lipoproteins are more potent than LTA with respect to immunostimulating activity^23^. However, it is unlikely that lipoproteins present in the Sa.LTA preparation were responsible for the immuno-stimulating activity observed in this study because of the following reasons. First, the LTAs used in this study were purified using hydrophobic interaction chromatography followed by anion-exchange chromatography, providing highly pure LTAs. It has been suggested that LTA prepared using only hydrophobic interaction chromatography is not sufficient to obtain highly pure LTA and an additional purification step, anion-exchange chromatography, is needed^40^. Second, unlike lipoproteins, Sa.LTA contains D-alanine moieties; however, in our study, after removal of D-alanine moieties the inhibitory potential on B cell proliferation was lost. Third, the inhibitory potential of Sa.LTA on LPS-induced B cell proliferation was also observed with Sa.LTA purified from a lipoprotein-deficient *S. aureus* mutant strain. Finally, according to a previous report^41^ and our observation in this study, lipoproteins strongly elicit mitogenic B cell proliferation rather than inhibiting it, even though both LTA and lipoproteins are recognized by TLR2^20^. Although LTA and lipoproteins share their recognition receptor, TLR2, immunological characteristics such as cell proliferative responses are different. It has been well documented that lipoproteins are recognized by TLR2/1 or TLR2/6 heterodimers^42^ while it is obscure whether LTA interacts with TLR2 heterodimer or homodimer. TLR2 needs co-receptors such as CD36 or CD14 to assist cellular signaling^43,44^. Although the interaction of CD36 and CD14 with TLR2 ligands is not clearly elucidated, it is likely that both co-receptors interact differently with LTA or lipoproteins, leading to different B cell proliferation. Furthermore, Sa.LTA, but not lipoproteins, directly interacts with paired-Ig-like receptor B, which negatively regulates immune responses^45^, suggesting that interaction of the TLR2 ligands with various receptors shows different immunological consequences. Furthermore, similar to our observation, *S. aureus* lipoproteins significantly induced cell proliferation in both human and murine B cells whereas Sa.LTA did not induce human or murine B cell proliferation^46^.

It can be assumed that LTA indirectly and directly acts on the inhibition of B cells. We described that Sa.LTA interacted with accessory cells such as macrophages and dendritic cells and more strongly inhibited splenocyte proliferation compared with the proliferation in isolated B cells. In addition, Sa.LTA-mediated inhibitory effect is associated with decreased ERK phosphorylation. Our study demonstrated that Sa.LTA decreased LPS-induced ERK activation in splenocytes and B cells. Moreover, when ERK signaling was blocked in splenocytes, the proliferative ability of LPS on B cells was diminished. This is in agreement with previous studies showing that transient activation of ERK signaling is not involved in cell-cycle entry, whereas sustained ERK activation is crucial for cell proliferation including B cells^47,48^. The activation of TLR2 heterodimer such as TLR2/1 heterodimer induces ERK1/2 activation^49^. In contrast, when TLR2 was solely blocked, ERK1/2 activation was decreased^50^. Unlike lipopeptides such as Pam3CSK4, which interacts with TLR2/1 heterodimer, LTA is not associated with either TLR2/1 or TLR2/6 heterodimer^20^, suggesting that LTA may inhibit ERK1/2 activation that is different from lipoproteins. Therefore, ERK plays a critical role in the inhibitory effect of Sa.LTA on LPS-induced B-cell proliferation.

Our findings also demonstrated that Sa.LTA did not directly affect the inhibition of LPS-induced B cell proliferation, but had an indirect effect through other cell types such as macrophages and dendritic cells. This observation is in agreement with previous studies showing that polyclonal B cell expansion can be induced by splenic macrophages or T cells^25^. Although it was not apparently described that the proliferation and differentiation of marginal zone or follicular B cells were strongly dependent on the splenic macrophages or T cells, the depletion of macrophages and T cells abolishes the capacity of proliferation and differentiation of splenic B cells^51,52^. Furthermore, a recent report also suggested that splenic macrophages are necessary for the B cell proliferation^53^ and stimulating factors such as a proliferation-inducing ligand from macrophages and dendritic cells potentiated human B cell proliferation^54^.

However, there are differences in the molecular mechanisms underlying the inhibitory activity of Sa.LTA. For example, previous studies have shown that secreted IL-10 effectively suppresses B cell proliferation *in vitro*^11^ and TGF-β1 inhibits LPS-mediated B cell responses to immunoglobulin production and cell proliferation^55^. In contrast, we found that those cytokines were not enhanced by Sa.LTA in our experimental settings. Furthermore, we also observed that the induction of B cell survival factors, such as IL-4, IL-5 or BAFF, were not regulated by Sa.LTA (data not shown), indicating that these survival factors did not play a role in the inhibition of LPS-induced B cell proliferation. In addition, up-regulation of perforin and granzyme in regulatory T cells was not seen in the presence of Sa.LTA (data not shown) although granzyme and perforin from regulatory T cells are known to directly kill B cells^9^.

In summary, the results of our studies showed that Sa.LTA, even with its similar structural and immunological characteristics to LPS and LTA, could not polyclonally expand B lymphocytes. Instead, Sa.LTA antagonized LPS-induced B cell proliferation. However, how this unique role of Sa.LTA on B cell proliferation affects bacterial pathogenesis and host immune responses remains to be further elucidated. For example, Sa.LTA may interfere with the establishment of host immune responses as an immune evasion mechanism. Furthermore, growing evidence suggests that lymphomas arising from marginal zone B cells of extranodal organs such as spleen are associated with microbial infections including *Helicobacter pylori*, *Campylobacter jejuni* and hepatitis C virus^56^. A number of preclinical studies have shown possible mechanisms including the inhibition of Bruton tyrosine kinase pathway to alleviate B-cell lymphoproliferative disorders^57^. Although extensive studies are needed to elucidate the impact of bacterial LTA from non-pathogens on the treatment of B cell lymphomas, it would be conceivable that bacterial LTA may be one of the therapeutic agents for the abnormal lympoproliferative disorders.

## Materials and Methods

### Reagents

Pam2CSK4 and *E. coli* K12 LPS were purchased from InvivoGen (San Diego, CA, USA). *E. coli* O111:B4 LPS was obtained from Sigma-Aldrich (St. Louis, MO, USA). Antibodies used for flow cytometric analysis or Western blotting were purchased from BioLegend or Cell Signaling Technology (Beverly, MA, USA), respectively. Carboxyfluorescein diacetate succinimidyl ester (CFSE) and Alexa Fluor^®^ 546 monoclonal antibody labeling kits were obtained from Invitrogen (Grand Island, NY, USA). U0126 and JAK inhibitor were purchased from Calbiochem (La Jolla, CA, USA).

### Mice

C57BL/6 mice were purchased from Orient Bio (Seongnam, Korea). C57BL/6 mice lacking TLR2 were kindly provided by Prof. Shizuo Akira (Osaka University, Osaka, Japan). Animal ethics approval for all experiments was obtained from Seoul National University (SNU-160726-8).

### Preparation of bacterial LTAs

Highly-pure LTAs from *S. aureus* ATCC 29213, *S. pneumoniae* ATCC 27336, *B. subtilis* ATCC 6633 (American Type Culture Collection; Manassas, VA, USA), *L*. *plantarum* KCTC 10887BP (Korean Collection for Type Culture; Daejon, Korea), and lipoprotein-deficient mutant (Δ*lgt*) *S. aureus* RN4220 (kindly provided by Prof. Bok Luel Lee; Pusan National University, Busan, Korea), were prepared as described previously^19,58^. LTA is an amphiphile composed of a hydrophilic polysaccharide and a hydrophobic glycolipid^59^. Therefore, highly-pure and structurally-intact LTAs were extracted using a mild organic solvent, followed by serial chromatographies: a hydrophobic-interaction column and a subsequent ion-exchange column^19^. Sa.LTA, Bs.LTA and Lp.LTA are considered a glycerophosphate-type while Sp.LTA is a ribiotolphosphate-type, therefore, the two different types of LTA were prepared according to previous reports^19,58^. Furthermore, the structural intactness of purified LTA was determined using a high filed 900 MHz NMR instrument and MALDI-TOF/TOF MS^39^. De-alanylated or de-acylated Sa.LTA was prepared by incubating native Sa.LTA in 0.1M Tris-HCl or 0.5N NaOH, respectively. De-alanylation and de-acylation of Sa.LTA were confirmed using a thin layer chromatography (data not shown).

### Cell preparation

Splenocytes were isolated from mice by homogenizing the spleens and removing erythrocytes using RBC lysing buffer (Sigma-Aldrich). Resting B cells from single cell suspensions of splenocytes were purified by negative selection with a B cell isolation kit containing biotin-conjugated monoclonal antibodies against CD43, CD4, and Ter-119 (Miltenyl Biotech Inc., Auburn, CA, USA) according to manufacturer’s instruction. The purity of B cell preparation was over 95% as determined by flow cytometric analysis of the B cell surface marker, B220.

### [^3^H]-thymidine incorporation assay

Splenocytes were plated in 96-well U-bottom microplates and incubated with LPS and/or LTA for 72 h at 37 °C in a 5% CO_2_-humidified incubator. Then, the cells were pulsed with 0.5 μCi/well of [^3^H]-thymidine for the last 6 h of culture periods. Quantification of [^3^H]-thymidine incorporation was measured with a microplate scintillation counter (Packard Bioscience, Meriden, CT, USA).

### Flow cytometric analysis

After cells were incubated with 2 μM CFSE in PBS at 37 °C for 20 min, CFSE-labeled cells were stimulated with indicated stimuli for 72 h. Then, the cells were stained with anti-CD45R/B220 antibody. B cell proliferation was determined by monitoring CFSE levels of the B220-positive cell population using a FACSCalibur flow cytometer with CellQuest software (BD Biosciences; San Jose, CA, USA). To examine TLR2 expression on each cell type of splenocytes, cells were stained with anti-TLR2 antibody on CD3^+^, B220^+^, CD11c^+^, or F4/80^+^ cells. To examine Sa.LTA binding ability to each cell type of splenocytes, the cells were stained with Alexa Fluor^®^ 546-conjugated Sa.LTA on CD3^+^, B220^+^, CD11c^+^, or F4/80^+^ cells. After staining, TLR2 expression or Sa.LTA binding ability was determined using a FACSCalibur flow cytometer.

### Enzyme-linked immunosorbent assay (ELISA)

Cell culture supernatants were subjected to the analysis of cytokine production using commercially-available ELISA kits (R&D Systems; Minneapolis, MN, USA).

### Western blot analysis

Western blot analysis was performed as described previously^60^. In brief, splenocytes or purified B cells were treated with LPS and/or Sa.LTA for 24 h and cell lysates were obtained using a lysis buffer (Proprep^TM^ protein extraction solution; iNtRon Biotechnology, Seongnam, Korea). The cell lysates were separated by 10% SDS-PAGE and transferred onto a polyvinylidene difluoride membrane (EMD Millipore, Bedford, MA, USA). After blocking with 5% skim milk, the membrane was incubated with antibodies specific to ERK or phosphorylated ERK followed by incubation with HRP-conjugated secondary antibodies. The immunoreactive bands were detected with SUPEX ECL solution (Neuronex, Pohang, Korea) and visualized with Chemi Doc MO (Bio-rad, Hercules, CA, USA). Band intensity was determined by densitometry using Image J software (National Institutes of Health, Bethesda, MD, USA).

### ***In vivo*** proliferation assay

Mice were injected *i*.*p*. with 50 μg of *E. coli* O111:B4 LPS and/or 300 μg of Sa.LTA for 48 h. Spleens were aseptically removed, weighed, and dispersed as single cells as described above. Subsequently, the cells were stained with anti-CD45R/B220 antibody and the B220-positive cell population was analyzed with flow cytometry, as described above.

### ***Ex vivo*** proliferation assay

At 24 h after mice were injected *i.p*. with 100 μg of Sa.LTA, the spleens were aseptically removed and single cell suspensions were prepared. The cells were CFSE-labeled and stimulated with LPS (0.1 μg/ml) for an additional 72 h. Following incubation, the cells were stained with anti-CD45R/B220 antibody and the B220-positive cell population was analyzed with flow cytometry, as described above.

### Statistical analysis

The mean value ± standard deviation was determined for each treatment group from independent three experiments. Treatment groups were compared to appropriate control(s) and statistical significance was determined using Dunnett’s two-tailed *t*-test. Differences were considered significant when *P* < 0.05 (*).

## Electronic supplementary material

Supplement information
