# Supplementary material for: Staphylococcal LTA antagonizes the B cell-mitogenic potential of LPS
Source: Sci Rep. 2018 Jan 24;8:1496. doi: 10.1038/s41598-018-19653-y (PMC5784022; doi:10.1038/s41598-018-19653-y)
Supplement: Supplementary file 1 — Supplement information [file 41598_2018_19653_MOESM1_ESM.pdf]

**Staphylococcal LTA antagonizes the B cell-mitogenic potential of LPS**

**Seok-Seong Kang[a], Sun Kyung Kim[b], Jung Eun Baik[b], Eun Byeol Ko[b], Ki Bum Ahn[b] Cheol-Heui Yun[c], Seung Hyun Han[b, *]**

[a]Department of Food Science and Biotechnology, Dongguk University-Seoul, Goyang 10326, Republic of Korea

[b]Department of Oral Microbiology and Immunology, DRI, and BK21 Plus Program, School of Dentistry, Seoul National University, Seoul 08826, Republic of Korea

[c]Department of Agricultural Biotechnology and Research Institute for Agriculture and Life Sciences, Seoul National University, Seoul 08826, Republic of Korea

*Corresponding author: shhan-mi@snu.ac.kr (S.H. Han)

## SUPPLEMENTARY FIGURES

**(a)**

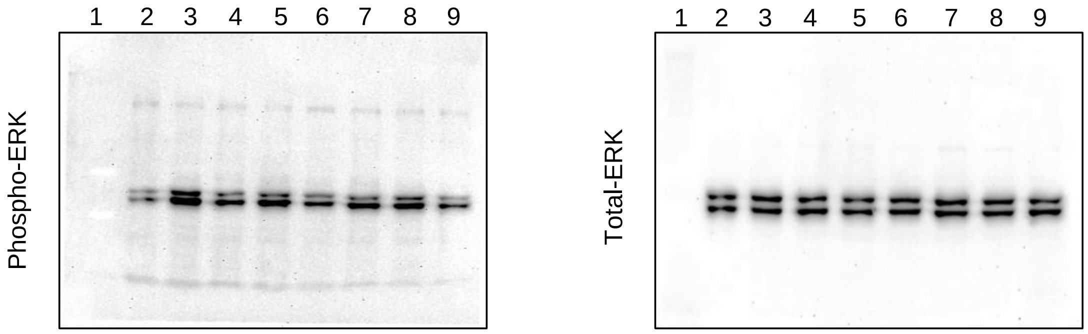


**(b)**

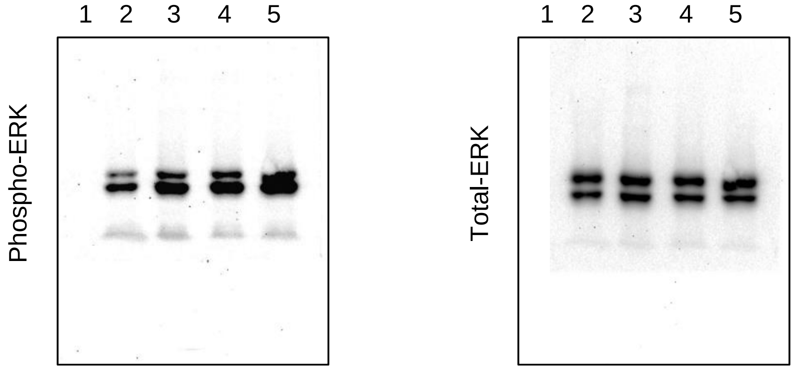


**Figure S1.** Complete blots with the indicated antibodies presented in the figure 4 of the manuscript. Panel **(a)** corresponds to blots of splenocytes (lane 2 to 5) and purified B cells (lane 6 to 9). Panel **(b)** corresponds to blots of splenocytes. For the panels **(a)**, lane 1: pre-stained protein ladder; lane 2: splenocytes without treatment; lane 3: splenocytes treated with LPS (0.1 μg/ml); lane 4: splenocytes treated with Sa.LTA (50 μg/ml); lane 5: splenocytes treated with LPS (0.1 μg/ml) and Sa.LTA (50 μg/ml); lane 6: purified B cells without treatment; lane 7: purified B cells treated with LPS (0.1 μg/ml); lane 8: purified B cells treated with Sa.LTA (50 μg/ml); lane 9: purified B cells treated with LPS (0.1 μg/ml) and Sa.LTA (50 μg/ml). For the panels **(c)**, lane 1: pre-stained protein ladder; lane 2: splenocytes without treatment; lane 3:

splenocytes treated with LPS (0.1 μg/ml) and Sa.LTA (50 μg/ml); lane 4: splenocytes pre-treated with Jak inhibitor (0.1 μM) followed by treatment with LPS (0.1 μg/ml) and Sa.LTA (50 μg/ml); lane 5: splenocytes pre-treated with Jak inhibitor (0.5 μM) followed by treatment with LPS (0.1 μg/ml) and Sa.LTA (50 μg/ml).
